# Aging modulated by the *Drosophila* insulin receptor through distinct structure-defined mechanisms

**DOI:** 10.1093/genetics/iyaa037

**Published:** 2021-01-05

**Authors:** Rochele Yamamoto, Michael Palmer, Helen Koski, Noelle Curtis-Joseph, Marc Tatar

**Affiliations:** Department of Ecology and Evolutionary Biology, Brown University, Providence, RI, USA

**Keywords:** insulin receptor, longevity, aging, reproduction, Drosophila, kinase insert domain

## Abstract

Mutations of the *Drosophila melanogaster* insulin/IGF signaling system slow aging, while also affecting growth and reproduction. To understand this pleiotropy, we produced an allelic series of single codon substitutions in the *Drosophila* insulin receptor, *InR*. We generated *InR* substitutions using homologous recombination and related each to emerging models of receptor tyrosine kinase structure and function. Three mutations when combined as trans-heterozygotes extended lifespan while retarding growth and fecundity. These genotypes reduced insulin-stimulated Akt phosphorylation, suggesting they impede kinase catalytic domain function. Among these genotypes, longevity was negatively correlated with egg production, consistent with life-history trade-off theory. In contrast, one mutation (*InR*^353^) was located in the kinase insert domain, a poorly characterized element found in all receptor tyrosine kinases. Remarkably, wild-type heterozygotes with *InR*^353^ robustly extended lifespan without affecting growth or reproduction and retained capacity to fully phosphorylate Akt. The *Drosophila* insulin receptor kinase insert domain contains a previously unrecognized SH2 binding motif. We propose the kinase insert domain interacts with SH2-associated adapter proteins to affect aging through mechanisms that retain insulin sensitivity and are independent of reproduction.

## Introduction

Insulin/insulin growth factor (insulin/IGF) signaling provides a powerful avenue to study aging. Early work extended lifespan through mutations of the insulin-like receptors *daf-2* of *C. elegans* and *InR* of *Drosophila* (Kenyon *et al.* 1993; Kimura *et al.* 1997; Tatar *et al.* 2001; Altintas *et al.* 2016). These invertebrates have single insulin-like receptors that simultaneously regulate the metabolic and growth functions of mammalian insulin receptor (IR) and insulin growth factor receptors (IGFR) (Finch and Ruvkun 2001; Tatar *et al.* 2003). As in mammals, Daf-2 and InR are stimulated by insulin-like peptides to induce intracellular signals through Akt (also called Protein kinase B), Ras (Proto-oncogene protein P21), and TOR (Target of rapamycin) (Teleman 2010), which collectively mediate transcription factors (Lin *et al.* 1997; Hsu *et al.* 2003) and cellular metabolism (Jia *et al.* 2004; Burkewitz *et al.* 2014; Post *et al.* 2018). These pathways ultimately assure survival through mechanisms that include translation rate, autophagy, mitochondrial uncoupling, REDOX, genomic stability, and lipogenesis (Honda and Honda 1999; Murphy *et al.* 2003; Mostoslavsky 2008; Perez and Van Gilst 2008; Zarse *et al.* 2012; Bai *et al.* 2013). Altered insulin/IGF function also affects aging in mammals. With some variation in outcome, knockdown of IR, IGF1R, and insulin receptor substrates (IRS1, IRS2) is associated with retarded aging in mice (Blüher *et al.* 2003; Taguchi *et al.* 2007; Selman *et al.* 2008; Xu *et al.* 2014; Merry *et al.* 2017). In humans, polymorphisms of *IGF1R* and the transcription factor *FOXO3A* are associated with exceptional lifespan (Suh *et al.* 2008; Bao *et al.* 2014). Overall, insulin/IGF signaling integrates cells, tissue and physiology to control adult survival and lifespan.

The breadth of insulin/IGF function challenges our ability to understand how it affects aging. Insulin/IGF signaling simultaneously regulates many traits including growth, metabolism, cell proliferation, differentiation, Dauer/diapause, and reproduction (Kido *et al.* 2001; Bartke *et al.* 2013; Ashpole *et al.* 2017; Ewald *et al.* 2018). Furthermore, these traits are mediated by a single insulin-like receptor in *C elegans* and *Drosophila* while these invertebrates produce many insulin-like ligands (Brogiolo *et al.* 2001; Pierce *et al.* 2001; Gronke *et al.* 2010). The insulin-like receptor is a molecular switch-board taking many incoming calls and routing each to distinct signaling destinations. Broadly, understanding such pleiotropy is a longstanding problem in receptor tyrosine kinase biology (Marshall 1995; Zinkle and Mohammadi 2018). In the biology of aging, a key aspect of pleiotropy involves trade-offs between lifespan and reproduction where alleles of a gene are favored that optimize the conflicting functions supporting these life-history traits (Tabatabaie *et al.* 2011; Maklakov *et al.* 2017). In this context, the mechanistic basis for how a protein such as the insulin-like receptor jointly controls lifespan and reproduction is unknown.

One approach to understand insulin/IGF receptor pleiotropy is to study alleles in cultured cells (Tazearslan *et al.* 2011; Hall *et al.* 2020), among polymorphic animals (Paaby *et al.* 2014), and even in humans with inherited insulin and IGF resistance (Klammt *et al.* 2011; Ardon *et al.* 2014). Insight is developed by relating the observed amino acid substitutions to receptor structure and function. In aging biology, this strategy was pioneered by work with *C. elegans daf-2* (Gems *et al.* 1998; Patel *et al.* 2008). About two dozen *daf-2* alleles extend lifespan, and variously also affect Dauer, fertility, and growth. Classes of these traits were defined based on where substitutions fell in the ectodomain relative to the kinase domain, but understanding how the substitutions jointly control traits was limited by the structural data of the time.

Structural analysis of receptor tyrosine kinases has advanced in recent years and we can now apply this to *Drosophila*. The *Drosophila* insulin-like receptor was initially identified in the lab of Rosen (Petruzzelli *et al.* 1986). EMS mutagenesis was subsequently used to generate a series of *InR* alleles that were viable as trans-heterozygotes yet otherwise recessive lethal (Azpiazu and Frasch 1993; Fernandez *et al.* 1995; Chen *et al.* 1996). Sequencing of several alleles identified substitutions in the kinase domain (Brogiolo *et al.* 2001). These genotypes variously reduced cell growth, fecundity, and receptor kinase activity (Chen *et al.* 1996; Brogiolo *et al.* 2001). In early work with the *Drosophila* insulin receptor, aging was slowed by one EMS-generated mutant (*InR*^E19^) when heterozygous over a P-element insertion allele (Tatar *et al.* 2001). Here, we characterize how other *InR* mutations from the original EMS collection affect aging using the method of quantitative complementation testing (QCT) (Flatt 2004; Geiger-Thornsberry and Mackay 2004). We then validate and extend these results by independently regenerating the putative substitutions through homologous recombination gene replacement in a separate genetic background and evaluate lifespan, growth, development rate, fecundity, and insulin sensitivity. Finally, recent advances provide new insights into the structure of activated human insulin receptors (Uchikawa *et al.* 2019; Chen *et al.* 2020) that we apply to understand how the *Drosophila* insulin-like receptor modulates aging.

We propose three conclusions. (1) *Drosophila* InR mediates aging in independent ways. One affects reproduction and thereby extends lifespan through reduced survival costs, the other affects mechanisms to assure longevity independent of reproduction. (2) The biochemical impact of substitutions within *InR* may be understood in a context of asymmetric transphosphorylation (Zinkle and Mohammadi 2018; Chen *et al.* 2020). Aging is slowed when one activated protomer contains a mutation that alters the quality or quantity of signaling. (3) Mutations slow aging by altering the receptor’s kinase activity or protein domain interactions. We identify a dominant substitution in the kinase insert domain (Locascio and Donoghue 2013) that robustly increases lifespan yet produces normal growth, high fecundity, and wild-type insulin sensitivity. This kinase insert domain contains a SH2 binding motif. We propose this site interacts with as yet to be identified adapter proteins to regulate longevity.

## Materials and methods

### EMS-induced mutations of InR

The Drosophila insulin/IGF-receptor *InR* is on the third chromosome. Lines with EMS-induced mutations in *InR*, balanced by the third chromosome inversion *InR*^+^*TM3*, were provided by M. Frasch (The Mount Sinai School of Medicine) (Azpiazu and Frasch 1993; Fernandez *et al.* 1995). We tested the ability of 17 EMS mutations to produce viable adults when complemented to the EMS-induced mutation *InR*^E19^ (provided by J. Jack, University of Connecticut) (Chen *et al.* 1996; Tatar *et al.* 2001). Nine Frasch alleles produced viable adults when complemented to *InR*^E19:^  *InR*^74^, *InR*^76^, *InR*^211^, *InR*^246^, *InR*^262^, *InR*^327^, *InR*^351^, *InR*^353^, and *InR*^373^. Subsequently, these nine alleles and the *InR*^E19^ allele were crossed six generations into the inbred *Samarkand I-236* (*Sam*) background using the balancer stock *Sam 1; Sam 2; InR*^+^*TM3*/*TM6B* (provided by T. Mackay, North Carolina State University).

### Wild-type InR^NC^ alleles

Iso-female lines were established from wild-type *Drosophila melanogaster* caught at the Raleigh Farmer’s Market (provided by T. Mackay, North Carolina State University) (De Luca *et al.* 2003). Single females from each line were crossed to males of *Sam 1; Sam 2; InR*^+^*TM3*/*TM6B* and backcrossed to extract third chromosomes balanced by *InR*^+^*TM3*. Eighteen chromosome extraction lines from this collection provided a sample of wild-type *InR* alleles (denoted *InR*  ^NC^ series). In addition, the *InR*^NC442^ line was used to normalize survival data across test blocks.

### Genotypes for quantitative complementation test

Quantitative complementation testing measures the phenotypic difference of one allele complemented to a standard hypomorph relative to when it is complemented to a standard wild-type allele (Long *et al.* 1996; Mackay and Fry 1996; Flatt 2004; Geiger-Thornsberry and Mackay 2004; Turner 2014). We made reciprocal crosses between *InR*^E19^ (using adult *InR*^E19^/*InR*^+^*TM3*) and the nine viable Frasch *InR* EMS-alleles (using *InR*^EMS^/*InR*^+^*TM3*), and between the 18 *InR*^NC^ wild-type alleles (using *InR*^NC^/*InR*^+^*TM3*). Offspring from reciprocal crosses were pooled to measure phenotypes. Phenotypes of every *InR*^EMS^/*InR*^E19^ and *InR*^NC^/*InR*^E19^ offspring were compared to a contemporaneous cohort of the allele when complemented by wild-type *InR* (*InR*^EMS^/*InR*^+^*TM3* or *InR*^NC^/*InR*^+^*TM3*).

#### Demographic quantitative complementation

Life tables (number of deaths per 2-day census period) were generated for adult offspring of each genotype (details in Supplementary Methods). Cohorts were initiated with three replicate demography cages of ∼150 newly eclosed adults at ∼1:1 sex ratio per genotype. In total, we assessed the lifespan of 16,057 males and 17,223 females. Each 2 days, food vials were changed and dead flies were removed and counted. Life tables collected in five test blocks (*j *=* *1–5) were made with data from replicate cages combined. Within each test block, we included the wild-derived line *InR*^NC442^ to normalized genotypes across blocks. One life table was generated for each *InR*^EMS^ allele and *InR*^NC^ allele expect for duplicates generated in independent blocks for *InR*^211(EMS)^, *InR*^74(EMS)^, and *InR*^353(EMS)^.

We used proportional hazard analysis (Parmer and Machin 1995) to conduct QCT with cohort life table data, executed in JMP (SAS Institute, Cary, NC). We quantified the proportional mortality difference (hazard coefficient *β_ij)_* within each block *j* for each *InR* allele *i* (EMS or NC) when complemented to *InR*^E19^ relative to when complemented to *InR^+^TM3*. In each block, we estimated $\beta_{NC442,j}$ and calculated the *adjusted mortality coefficient* for each *InR* allele as $\beta_{i}^{\mathrm{adj}}=\beta_{ij}-\beta_{NC442,j}$. Negative values of $\beta_{i}^{\mathrm{adj}}\;$indicate mortality rate is reduced in *InR^i^*/*InR*^E19^ relative to *InR^i^*/(*InR^+^TM3*); values near zero indicate there is no effect on mortality and values greater than zero indicate there is increased mortality. The transformation $\text{exp}\;\left|\beta_{i}^{\mathrm{adj}}\right|$ estimates the fold change in mortality risk (hazard ratio) induced by each *InR* allele complemented to *InR*^E19^ relative to when it is complemented to *InR^+^TM3*. Life expectancy was also standardized among blocks: for each block *j* we calculated the difference in life expectancy between *InR*^NC442^/*InR*^E19^ and *InR*^NC442^/(*InR^+^TM3*) denoted $\Delta E_{0}({\mathrm{InR}}_{j}^{NC442})$. The adjusted difference in life expectancy for each *InR* allele *i* is $\Delta E_{0,i}^{\mathrm{adj}}=\Delta E_{0,ij}-\Delta E_{0}{(\mathrm{InR}}_{j}^{NC442})$.

### Body size quantitative complementation

Head capsule width was measured from 20 females from each *InR*^EMS^ mutant and *InR*^NC^ wild-type allele when complemented to *InR*^E19^ and to *InR^+^TM3*. Adults were reared simultaneously on standard diet from vials seeded with 100 eggs, and collected across three replicate vials. The relative body size of each tested *InR^i^* allele was measured as (mean of *InR^i^*/*InR*^E19^)/(mean of *InR^i^*/*InR^+^TM3*).

### Homologous recombination InR alleles

To validate and extend the results of QCT, we focused on the EMS alleles *InR*^E19^, *InR*^211^, *InR*^74^, and *InR*^353^ based on their ability to reduce mortality, and *InR*^246^ as a contrast where QCT showed effects body size but not mortality. Ron Kohanski (Johns Hopkins University, personal communication) provided cDNA sequences for the *InR*^E19^, *InR*^74^, and *InR*^246^ alleles that respectively contained codon substitutions as Val*810*Asp, Ile*1543*Phe, and Val*1384*Met (Figure 3). Codon substitutions for the EMS alleles *InR*^353^ and *InR*^211^ were reported by Brogiolo *et al.* (2001) (Figure 3). In previous work as in our QCT, the causal effect of these substitutions has not been independently verified. Accordingly, we used ends-out homologous recombination gene replacement to regenerate each codon substitution in a coisogenic *w*Dah background. We made five single replacement lines (designated *InR*^E19(HR)^, *InR*^74(HR)^, *InR*^211(HR)^, *InR*^246(HR)^, *InR*^353^^*(*^^HR)^). We produced independent, replicate accessions for *InR*^E19(HR)^ (denoted *InR*^E19(HR)^-2, *InR*^E19(HR)^-14, *InR*^E19(HR)^-22) and for *InR^353^*^(HR)^ (denoted *InR*^353(HR)^-8, *InR*^353(HR)^-15, *InR*^353(HR)^-20). We replaced the native *w*Dah sequence with itself to produce the wild-type control *InR*^+(HR)^ (accession denoted “29B”). We generated a double substitution allele (*InR*^E19; 74(HR)^), and a null *InR* allele (*InR*^null(HR)^).

Detailed methods for the homologous recombination are provided in the Supplementary Materials. Targeting arms for *InR* cloned from *w*Dah (Staber *et al.* 2011) were inserted into the pW25.2 vector (Drosophila Genomics Resource Center, Bloomington, IN) and injected into *w*^1118^ embryos by Genetic Services (Sudbury, MA). Targeting arms contained single nucleotide substitutions to produce the desired amino acid replacements. Flies with the *white^+^* eye color marker mapped to the first or second chromosomes were used for homologous recombination. These lines were crossed to flies expressing flipase and the restriction enzyme *I-Sce1* to execute excision and linearization of the transgenic construct. Candidate insertion lines were selected based on *white^+^* mapped to chromosome 3. Chromosomes with replaced alleles were crossed to flies expressing *cre* recombinase to remove the *white^+^* marker. The *white^+^* marker was retained in an accession of the wild-type replacement allele to produce *InR*^null(HR)^. All replacement alleles were validated by sequencing.

### Phenotypes of InR homologous recombination alleles

#### Viability, development time, and adult size

Reciprocal crosses were made among all *InR*^(HR)^ alleles. Four females and males were housed for mating and to lay eggs over 24 h in replicate vials with yeast-supplemented standard media. We recorded stage of lethality (larvae, prepupae, or pupae) and the number of emerging adults each subsequent 24 h. Adult size (thorax length) was measured from 20 off-spring females of every viable cross.

#### Life table demography

Newly eclosed males and females were collected, transferred to bottles with fresh medium and kept at 25°C for 2 days (details in Supplementary Methods). Mated females were transferred to demography cages maintained at 25°C at an initial density of 125 females per cage. Three to five replicate cages were initiated for each genotype. Food was changed every two days, at which time dead flies were removed and counted. Life tables were constructed with genotype replicate cages combined for survival analysis. In total, we assessed the lifespan of 17,184 homologous recombination females.

#### Fecundity and ovariole number

Fifteen newly eclosed females of each genotype were placed with 15 *w*Dah males into three replicate demography cages, modified for egg collection (details in Supplementary Methods). For the following 15 days, egg plates were supplemented with yeast paste and changed twice a day at which time eggs were counted. Dead females were removed and counted daily. Daily fecundity (mean eggs per day) is the number of eggs summed across replicate cages per surviving female. Average fecundity is the mean of daily fecundity across the sample period. Ovariole number was measured from fifteen 10-day old females of each genotype. Ovaries were dissected in PBS, fixed with 4% paraformaldehyde, and scored for total number of ovarioles across both ovaries.

#### Insulin stimulation of pAkt

We evaluated the ability of IRs to induce phosphorylation of Akt from larval fat bodies stimulated by human insulin, as in Musselman *et al.* (2011). For each biological sample, wandering L3 larvae were rinsed in PBS, bisected in Schneider’s medium (Gibco Cat#21-720-001) and the anterior half everted. This tissue was transferred to Eppendorf tubes containing 500 µl Schneider’s medium with 0, 1, 2, or 5 µg/ml human insulin (Santa Cruz Biotechnology; Cat#SC-360248) and incubated at room temperature with agitation for 20 min. Samples were transferred to 75 µl of sample buffer containing phosphatase inhibitors, homogenized, and used for Western-blot (Invitrogen NUPage system). Drosophila specific phospho-Akt antibody (Ser505) and pan-Akt were obtaining from Cell Signaling Technology (PO_4_-Akt Cat#4054, pan-Akt Cat#4691). Blots were imaged and quantified with ImageLab (Bio-Rad).

### Data availability

#### Insulin receptor alignments and data access

Figure 3 and Supplementary Figure S1 provide amino acid sequence annotation of *Drosophila* InR, and human IR and IGF1R. We number the human IR as the mature long isoform of Ebina *et al.* (1985) (NCBI NP_000199.2). For cross-reference, the Human Genome Variation Society annotation includes an additional 27 a.a. localization sequence (Hall *et al.* 2020). The human IGF1R sequence is numbered as NCBI NP_000866.1. Throughout this manuscript we number *Drosophila InR* beginning at the first translation initiation site in *w*Dah wildtype (deposited in GenBank as accession MT_563159), which is +45 amino acids relative to the TIS reported by Fernandez *et al.* (1995) (NCBI NM_079712.6). The *w*Dah sequence also differs from that of Fernandez by an insertion/deletion (*w*Dah lacks H*144*) and the substitution H*146*Q. This H*144*/H*146* polymorphism segregates along latitudinal clines of *Drosophila* populations (Paaby *et al.* 2014). Supplementary Figures S4 and S5 provide the verified sequence alignments of the homologous recombination alleles.

#### Contents of Supplementary materials

Supplementary Methods contain detailed methods for demography, stocks, homologous recombination, and all PCR primers. Supplementary Materials contain figures for InR/hIR/hIGFR alignment (Supplementary Figure S1), eclosion time distribution (Supplementary Figure S2), and the verified sequence alignments of the homologous recombination alleles (Supplementary Figures S4 and S5). Supplementary Figure S3 provides conceptual diagrams of dimer complementation and a model for receptor domain function in aging. Tables in the Supplemental Materials provide survival statistics of the QCT (Supplementary Table S1), survival data for wild-type alleles in QCT (Supplementary Table S2), survival data of hemizygotes (Supplementary Table S3), and survival data of all HR alleles and accessions (Supplementary Table S4). 

Supplementary material is available at figshare DOI: https://doi.org/10.25386/genetics.13377023.

## Results

### Demographic quantitative complementation

**Figure 1:**
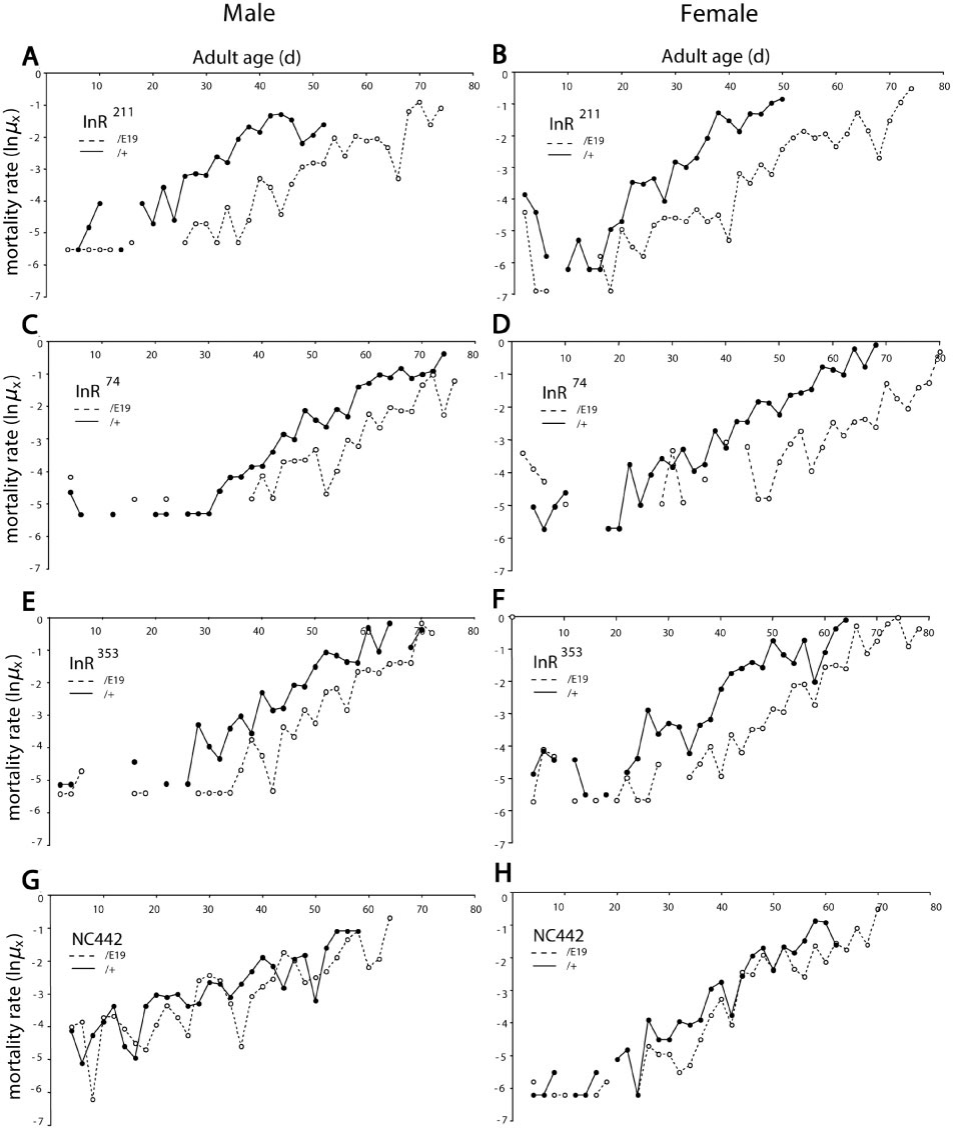
Mortality of EMS- and wild-type *InR* alleles in quantitative complementation test. Log mortality rate plotted from observed age-specific mortality (*q_x_*), ln*µ_x_* = ln(−ln(1 − *q_x_*)). Block 3 data (Supplementary Table S3): *InR*^allele^/*InR*^E19^ (dashed line), *InR*^allele^/(*InR*^+^, TM3 *Sb*) (solid line). N_0:_ initial cohort size combined genotypes. Cox proportional hazard analysis estimates mortality coefficient *β* for each allele as *InR*^allele^/*InR*^E19^ relative to *InR*^allele^/(*InR*^+^ TM3 *Sb*); *β*  <  1.0 indicates *InR*^allele^/*InR*^E19^ has reduced mortality, *P* < 0.0001 (survival statistics in Supplementary Table S1). (A, B) *InR*^211^: males (*N*_0_ = 484, *β* = −1.65, SE = 0.11); females (*N*_0_ = 712, *β* = −1.82, SE = 0.10). (C, D) *InR*^74^: males (*N*_0_ = 337, *β* = −0.98, SE = 0.12); females (*N*_0_ = 442, *β* = −1.68, SE = 0.13). (E, F) *InR*^353^: males (*N*_0_ = 394, *β* = −0.99, SE = 0.11); females (*N*_0_ = 560, *β* = −1.42, SE = 0.099). (G, H) NC442 third chromosome (wild-type *InR*): males (*N*_0_ = 443, *β* = −0.504, SE = 0.099); females (*N*_0_ = 571, *β* = −0.97, SE = 0.11).

**Figure 2:**
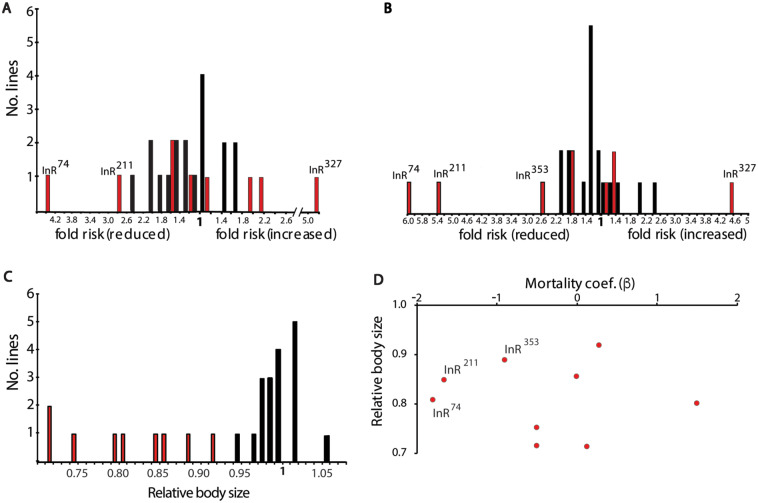
Distributions of allelic effects estimated from Quantitative Complementation Test comparing EMS- and wild-type InR alleles. (A) Males, (B) females; distribution of proportional hazard (fold risk of mortality) for nine EMS mutants (red bars) and 18 wild-type (NC series) alleles (black bars); estimated as exp(|β|). EMS *InR* alleles with significantly different mortality are labeled (Statistics in Supplementary Table S1). (C) Distribution of relative body size (head capsule width) among mutant (red) and wild-type (black) females. All mutants were smaller than the expected based on the distribution of wild-type alleles, *z*-test, *P* < 0.0007. (D) No significant correlation between relative female mortality (*β*) and relative body size among *InR* alleles assessed in QCT (*r*^2^ = 0.08, *P* = 0.85).

We used QCT to determine how the viable Frasch *InR* EMS alleles (*InR*^74^, *InR*^76^, *InR*^211^, *InR*^246^, *InR*^262^, *InR*^327^, *InR*^351^, *InR*^353^, and *InR*^373^) affect aging. Figure 1 illustrates mortality for several mutants complemented to *InR*^E19^ relative to when complemented to wild-type *InR* (*InR*^+^*TM3*) (Figure 1, Supplementary Table S1). Mortality rates in all cohorts increased exponentially once the death rate exceeded the lower limit of demographic power (Promislow *et al.* 1999). Life tables were generated in five blocks, with the natural wild-type allele *InR*^NC442^ measured in each to control for block effects (Supplementary Table S2). Relative to *InR*^NC442^ within each block, the EMS mutations variously reduced mortality (block adjusted *β_i_*^adj^ = -1.80), had little effect (*β_i_*^adj^  ≈ 0) or substantially increased mortality (*β_i_*^adj^ = 1.64) (Supplementary Table S1). Because *InR*^NC442^ is just one instance of a wild-type allele, we applied QCT to a collection of 18 wild-type *InR* alleles extracted from the North Carolina Market population (NC series) and evaluated how each EMS mutant performed relative to the wild-type distribution (Supplementary Table S1). Six EMS *InR* mutants did not significantly reduce mortality relative to wild-type alleles (*InR*^76^, *InR*^246^, *InR*^262^, *InR*^273^, *InR*^327^, and *InR*^351^), while *InR*^327^ strongly increased mortally. *InR*^74^ and *InR*^211^ significantly reduced mortality in males and females. The *InR*^353^ allele reduced female mortality but not significantly so relative to the wild-type distribution. The transformation $\text{exp}\;\left|\beta_{i}^{\mathrm{adj}}\right|$ estimates fold change in mortality: *InR*^74^ and *InR*^211^ alleles reduced male and female mortality 2.6- to 6-fold, while *InR*^353^ reduced female mortality about 2-fold (Figure 2, A and B).

### Body size quantitative complementation

To describe the allelic effects on female body size, we quantified the ratio of each EMS allele complemented to *InR*^E19^ relative to *InR*^+^*TM3*. All EMS *InR* alleles significantly reduced body size, with relative effects ranging from 0.9 to less than 0.75 (Figure 2C). Among the EMS *InR* alleles, relative body size does not correlate with a change in mortality (Figure 2D).

### Homologous recombination InR alleles

Quantitative complementation testing has limitations: it only measures recessive allelic effects; it confounds epistatic interactions with potential co-segregating mutations; the tested alleles were not derived from the wild-type *InR* (*InR*^+^*TM3*) used for complementation; each tested *InR* may contain unidentified substitutions; allelic effects are relative rather than absolute; and the measured effect of *InR* is confounded by a deleterious effect of the TM3 balancer chromosome. Our newly generated series of homologous recombination *InR* alleles resolves these issues.

We evaluated viability, eclosion time, and adult size for all allele combinations (Figure 4, A and B, Supplementary Figure S2). Every mutation had normal size and development time when heterozygous with *InR^+^*^(HR)^. All mutations were homozygous lethal except for rare escapes in the case of *InR*^E19(HR)^/*InR*^E19(HR)^. As expected from our QCT, all kinase domain mutations (*InR*^74(HR)^, *InR*^211(HR)^, *InR*^246(HR)^, and *InR*^353^^*(*^^HR)^) produced small adults with delayed development when heterozygous with *InR*^E19(HR)^ (an ectodomain mutation). Several kinase domain mutations were viable as trans-heterozygotes: *InR*^74(HR)^/*InR*^353(HR)^ and *InR*^74(HR)^/*InR*^211(HR)^, and these adults were small and developed slowly.

### Demography of InR hemizygotes

Insulin receptors are preformed dimers assembled with protomers translated from either allele (Supplementary Figure S3). Because homozygotes of mutant alleles are inviable, we infer homodimeric mutant receptors are nonfunctional. Viable mutant trans-heterozygotes must produce functional heterodimers, but they will also make fewer functional dimers because half of all potential receptors are mutant homodimers. To determine if simply having fewer functional receptors extends longevity, we evaluated the survival of hemizygote *InR^+^*^(HR)^*/InR*^null(HR)^ adults, which produce about 25% less *InR* mRNA (quantitative PCR in Supplementary Table S3). These adults increased life expectancy between 2 and 4 days when tested in replicate trials (Figure 5; significant only in Trial 2; Supplementary Table S3). This difference provides an approximate benchmark: we may infer a genotype affects receptor *function* when its gain in longevity is more than that caused by *numerical* loss of receptors modeled in *InR* hemizygotes.

### Demography of InR homologous recombination adults

The heterozygote *InR*^E19(HR)^/*InR*^+(HR)^ does not extend lifespan (Figure 6A): median life expectancy averaged among *InR*^E19(HR)^ accessions was 43.3 days, compared to 44 days for wildtype (Supplementary Table S4). Likewise, the intracellular kinase domain alleles *InR*^74(HR)^, *InR*^211(HR)^, and *InR*^246(HR)^ when heterozygous with *InR^+^*^(HR)^ did not extend lifespan more than expected from *InR* hemizygotes (Figure 6, B and C; Supplementary Table S4). In contrast, in replicated trials with independent accessions, *InR*^+(HR)^/*InR*^353(HR)^ increased life expectancy on average 12.5 days by decreasing mortality about fourfold (Figure 6, C and D; Supplementary Table S4).

In QCT, kinase domain alleles reduced mortality when complemented to *InR*^E19^ relative to when complemented to *InR*^+^. From the homologous recombination alleles, we now estimate the absolute allelic effect of each mutation. Across replicate trials, the genotypes *InR*^74(HR)^/*InR*^E19(HR)^ and *InR*^211(HR)^/*InR*^E19(HR)^ extended lifespan 6–14 days (Figure 6, E and F). We also find the kinase domain heterozygote *InR*^74(HR)^/*InR*^211(HR)^ extended lifespan (Figure 6, E and F). In every case, these longevity benefits exceeded that of the *InR* hemizygote (Supplementary Table S4).

Because the trans-heterozygote *InR*^E19(HR)^/*InR*^74(HR)^ slowed aging, we asked if the mutations of these individual alleles extend lifespan when the substitutions were on the same protomer. They did not: *InR*^E19,74(HR)^ had little effect on survival when heterozygous over a wild-type allele (Figure 6B). We likewise tested *InR*^E19,74(HR)^ when heterozygous with *InR*^211(HR)^. These adults extended longevity to the same extent as trans-heterozygote *InR*^74(HR)^/*InR*^211(HR)^ (Figure 6E). Longevity is only extended when the substitutions of these two alleles occur on opposing protomers.

In QCT, the *InR*^246^ allele did not slow aging although it reduced body size. These observations are confirmed with *InR*^246(HR)^/*InR*^E19(HR)^: adults were small (Figure 4) but not long-lived (Figure 6, G and H). However, the survival plot of *InR*^246(HR)^/*InR*^E19(HR)^ intersects that of wildtype, suggesting the mutant genotype may have high age-independent mortality which obscures a beneficial effect of reduced demographic aging.

While *InR*^353(HR)^ extended lifespan when heterozygous with wildtype (10–16 days), *InR*^353(HR)^ also slowed aging when heterozygous with *InR*^E19(HR)^ (14–15 days). When heterozygous with *InR*^74(HR)^, *InR*^353(HR)^ extended lifespan 21–22 days, an effect caused by decreasing age-dependent mortality sixfold (Figure 6, G and H; Supplementary Table S4).

The homologous recombination alleles validate inferences from QCT but now measure absolute effects and are tested in an independent genetic background. We describe new trans-heterozygous genotypes that extend lifespan (*InR*^74(HR)^/*InR*^211(HR);^  *InR*^74(HR)^/*InR*^353(HR)^; *InR*^E19(HR)^/*InR*^353(HR)^) and document a dominant longevity benefit conferred by *InR*^353(HR)^ (*InR*^+(HR)^/*InR*^353(HR)^).

### Fecundity

Drosophila insulin signaling modulates fecundity (LaFever and Drummond-Barbosa 2005; Hsu *et al.* 2008). Because reproduction is a common trade-off with survival (Flatt 2011), we determined how the *InR* alleles affect egg production (Figure 7, A and B). Fecundity was similar among wildtype, hemizygote, and *InR*^E19(HR)^/*InR*^246(HR)^ females. Fecundity was reduced in all long-lived trans*-*heterozygotes. In contrast, long-lived *InR^+^*^(HR)^/*InR*^353(HR)^ females produced *more* eggs than wildtype. The elevated fecundity of *InR^+^*^(HR)^/*InR*^353(HR)^ could arise because these females have more ovarioles (subunits of the ovary) or produce more eggs per ovariole. We therefore measured ovariole number for all genotypes and regressed this trait against daily egg production. Fecundity positively associates with ovariole number with a slope less than one (Figure 7C): differences in daily fecundity derive in part from differences in the rate of egg production within ovarioles. We therefore regressed genotype lifespan against egg production per ovariole (eggs/day/ovariole), treating the *InR*^353(HR)^ allele as a covariate (Figure 7D). A striking pattern emerges. Egg production per ovariole negatively associates with life expectancy (*N* = 8, *P *=* *0.06), but the regression intercept for genotypes with one *InR*^353(HR)^ allele is ∼12 days greater than genotypes without this allele (*P *=* *0.013). The *InR*^353(HR)^ allele confers longevity that is additive to and independent of how reproduction associates with survival.

### Kinase activity of InR homologous recombination alleles

The *InR*^353(HR)^ substitution (R*1466*C) lies in the kinase insert domain, a region of receptor tyrosine kinases thought to modulate substrate interaction rather than receptor kinase activity (Locascio and Donoghue 2013). We therefore assessed Akt phosphorylation of fat body stimulated with insulin. As expected, pAkt was strongly induced in tissue from wild-type flies (Figure 7E). Fat body from the hemizygote *InR^+^*^(HR)^/*InR*^null(HR)^ was moderately insulin resistant. Fat body from trans*-*heterozygotes involving *InR*^E19(HR)^, *InR*^74(HR)^, *InR*^246(HR)^, and *InR*^211(HR)^ induced little pAkt; they were insulin resistant. In contrast, fat body from *InR^+^*^(HR)^/*InR*^353(HR)^ induced wild-type levels of pAkt. The *InR*^353(HR)^ allele dominantly extends lifespan without loss of insulin-stimulated kinase activity.

## Discussion

Mutations of insulin/IGF receptors are thought to slow aging and reduce growth because they diminish the total amount of insulin signaling. Alternatively, mutations of the IR may alter protein-receptor interactions in specific domains that control particular phenotypes, as proposed for receptor tyrosine kinases in general by Zinkle and Mohammadi (2018). Here, we explore these ideas to explain how *Drosophila InR* alleles modulate aging, growth, and reproduction.

### Alleles in the quantitative complementation test

We used QCT to identify EMS *InR* mutations that slow aging. Three alleles reduced mortality more than expected from a sample of natural wild-type alleles. We also quantify how alleles affect growth. While all alleles significantly reduced adult size, there was no association between relative size and relative mortality among genotypes.

As noted, QCT has caveats (Flatt 2004; Turner 2014). The EMS-derived *InR* mutations do not share a common genetic background. Epistatic interactions with unknown second site mutations on the third chromosome could affect traits attributed to *InR*. QCT only estimates recessive effects of the *InR* mutations, and we could not evaluate complementation besides with *InR*^E19^. Unidentified polymorphisms within each *InR* locus might influence the complementation. Finally, QCT measures relative rather than absolute allelic effects, and these relative effects were confounded by how the balancer haplotype itself reduced lifespan.

To address these issues, we used ends-out homologous recombination to reconstitute the putatively causal *InR* substitutions of selected EMS alleles. These new alleles provide single, defined substitutions derived from a common wild-type progenitor in a controlled genetic background. We produced five homologous recombination (HR) alleles (*InR*^74(HR)^, *InR*^211(HR)^, *InR*^246(HR)^, *InR*^353(HR)^, *InR*^E19(HR)^), an HR-derived wild-type allele, a null allele, and a *cis*-allele with the *E19* and *74* substitutions upon the same protomer (*InR*^E19,74(HR)^).

### Receptor quantity moderately affects longevity

InR is a dimeric transmembrane receptor. Flies with two wild-type alleles generate normal protomers so that every dimer is functional. On the other hand, trans-heterozygotes produce some functional dimers with protomers of complementing alleles, as well as nonfunctional homodimers from protomers of each single mutant allele (Supplementary Figure S3). Thus, the lifespan of trans-heterozygote adults may be slowed simply because they have fewer functional receptors. To test this idea, we studied the hemizygote *InR*^+(HR)^/*InR*^null(HR)^. This genotype reduced *InR* mRNA about 25% and presumably this reduced the total number of receptors. Hemizygotes moderately impaired phosphorylation of Akt and only modestly affected longevity. The number of receptors in these hemizygotes is expected to be similar to that of recessive lethal alleles when heterozygous with wildtype (*InR*^+(HR)^/*InR*^E19(HR)^, *InR*^+(HR)^/*InR*^74(HR)^, *InR*^+(HR)^/*InR*^211(HR)^) due to their loss of mutant-mutant dimers (Supplementary Figure S3). Yet, those adults have normal longevity. Together these data suggest that genotypes with robustly extended lifespan either further reduce the quantity of receptors, or alter the quality or quantity of signaling from their functional receptors.

### InR^E19(HR)^: extracellular fibronectin domain

**Figure 3:**
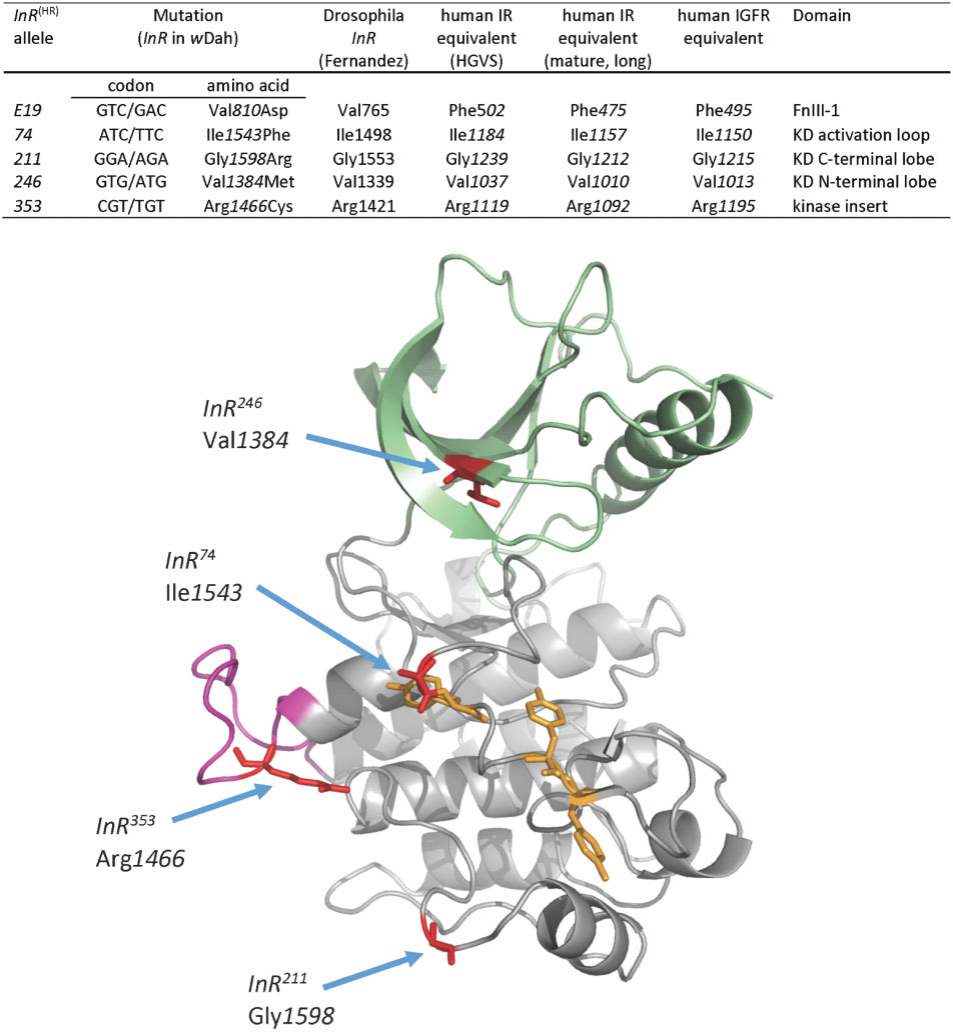
Sites, nomenclature, and structural location of InR alleles generated by homologous recombination. Amino acids numbered from the translation initiation site of the *w*Dah stock used as the progenitor wild-type *InR* in homologous recombination (GenBank accession MT_563159). This TIS is 45 additional N-terminal amino acids from the TIS reported in Fernandez *et al.* (1995). Human insulin receptor sequence is numbered following the nomenclature of the Human Genome Variation Society (HGVS; http://www.hgvs.org/rec.html) and based on the mature, long-form type IR cDNA of Ebina *et al.* (1985). The human IGF1R amino acid sequence is based on NCBI Reference Sequence: NP_000866.1. KD: kinase domain. FnIII: extracellular fibronectin domain III. Ribbon model of kinase domain (based on hIR), indicating conserved sites of amino acid substitutions (red). N-terminal lobe: green; C-terminal lobe: gray; kinase insert domain: magenta; Tyrosine residues of the activation loop: yellow.

The *InR*^E19(HR)^ substitution Val*810*Asp occurs in a linker sequence between the L2 and FnIII-1 ectodomains, corresponding to Phe*475* of human IR and to Phe*495* of human IGF-1R (Figure 3; Supplementary Figure S1). Insulin induces a hinge motion in this linker to swing the fibronectin domain inward, bringing together the intracellular kinase domains of each protomer (Gutmann *et al.* 2018; Scapin *et al.* 2018; Uchikawa *et al.* 2019). This induced proximity permits asymmetric kinase transphosphorylation, a non-reciprocal process whereby the intrinsic kinase activity of one protomer phosphorylates the opposing activation loop (A-loop) (Zinkle and Mohammadi 2018; Chen *et al.* 2020; Ferguson *et al.* 2020). The Val*810*Asp substitution of *Drosophila* InR will destabilize the fibronectin domain movement (Uchikawa *et al.* 2019) and thus prevent transphosphorylation of its associated intracellular kinase (Chen *et al.* 2020). The *InR^+^*^(HR)^*/InR^E19^*^(HR)^ genotype has wild-type traits because the Val*810*Asp protomer retains its ability to transphosphorylate the complementing wild-type kinase domain, which is sufficient for full receptor kinase function.

**Figure 4:**
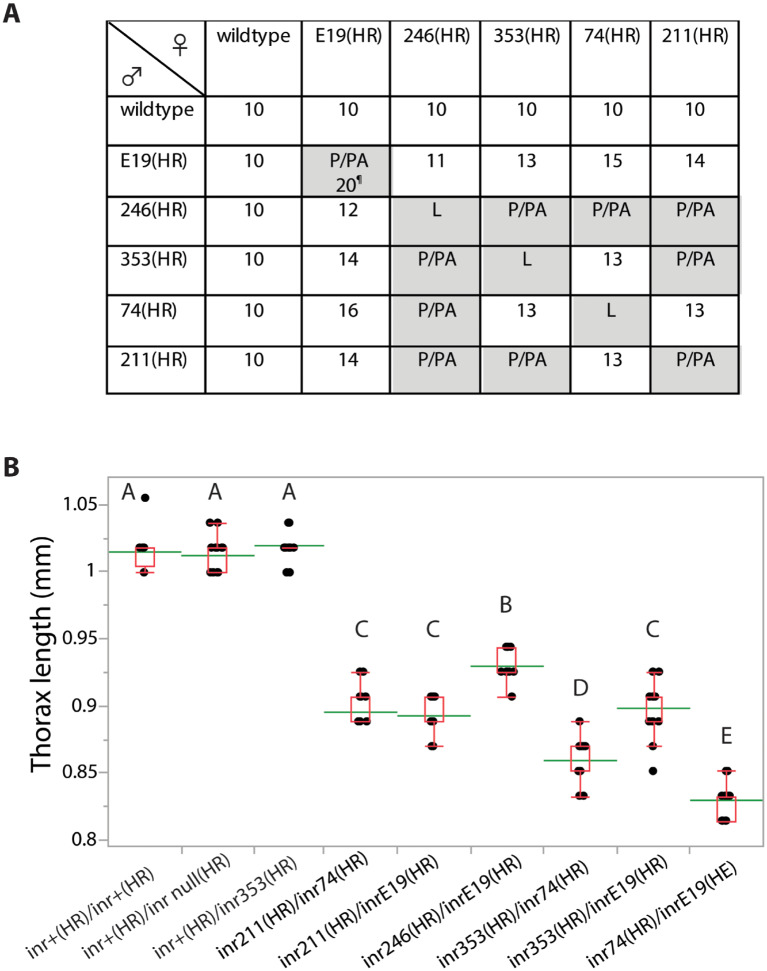
Growth characteristics of homologous recombination *InR*^(HR)^ alleles. (A) Development time (days to first eclosion), or stage of lethality for all allele combinations with reciprocal crosses. Lethality at first or second stage larvae (L), late pupae (P) or pharate adult (PA). ¶: *InR*^E19(HR)^/*InR*^E19(HR)^ produce few minute adults at 20 days, but otherwise were pupal/pharate lethal. (B) Adult size (thorax length, mm) of *InR*^(HR)^ alleles across genotypes. Means, std. dev. and range shown, *N* = 20 females each genotype. Means not connected by same letter are significantly different (one-way ANOVA, Tukey–Kramer HSD comparison, *P* < 0.05).

**Figure 5:**
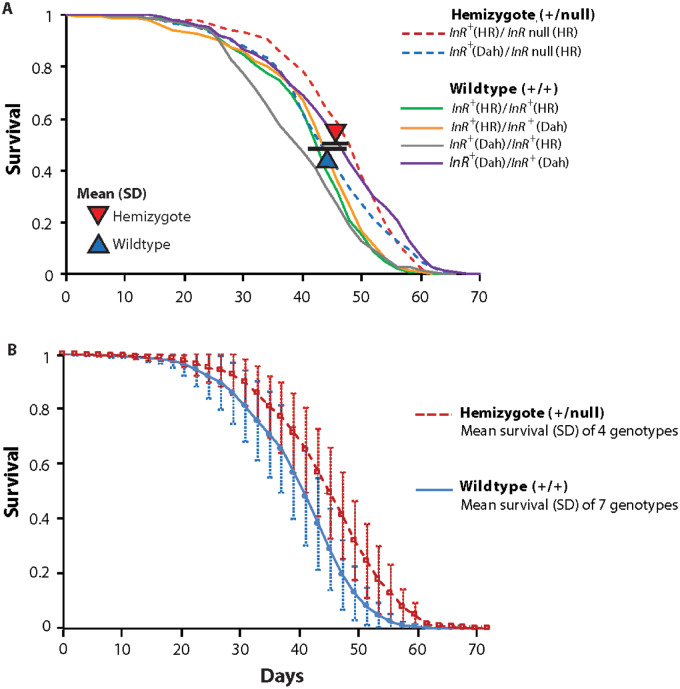
Impact of *InR* hemizygosity upon adult survival. (A) Trial 1. Two hemizygotes (*InR*^null(HR)^/*InR*^+(HR)^ and *InR*  ^null(HR)^*/InR^+^*^Dah^) compared to four wild-type (generated from reciprocal, pairwise combinations of *InR*  ^+(HR)^ and *InR*  ^+Dah^ alleles). Triangles indicate the average of median lifespan of the genotype group (bars: standard deviation). Hemizygotes are 2 days longer lived, arising from lower proportional hazard mortality (*β* = −0.14, *χ*^2^ = 36.4, *P* < 0.0001). (B) Trial 2. Four hemizygotes compared to seven wildtypes; genotype details in Supplementary Table S3. Survival plots show mean values among genotypes within the genotype class, with standard deviation (per census interval). Hemizygotes are 4 days longer lived, arising from lower proportional hazard mortality (*β* = −0.25, *χ*^2^ = 275.1, *P* < 0.0001).

**Figure 6:**
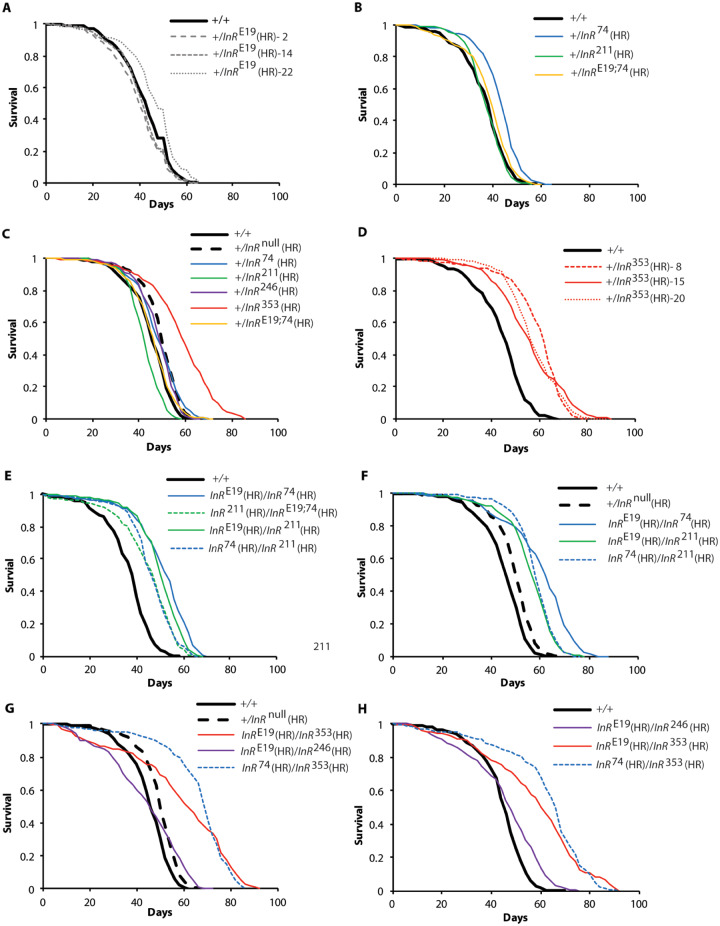
Survival of homologous recombination *InR* alleles. Females. Plots represent independent trials except for repeated controls shown in (B and E), and in  (C,  F, and G). In all trials, wild-type and null allele controls were homologous recombinant *InR*^+(HR)^ and *InR*^null(HR)^ respectively. (A) Wild-type heterozygotes made with three accessions of ectodomain mutant *InR*^E19(HR)^. (B) Wild-type heterozygotes made with mutants of the kinase domain A-loop (*InR*^74(HR)^) and C-terminal lobe (*InR*^211(HR)^). (C) Wild-type heterozygotes made with mutants of kinase domain N-terminal lobe (*InR*^246(HR)^), A-loop, KID (*InR*^353(HR)^), and C-terminal lobe; hemizygotes shown for contrast. (D) Wild-type heterozygotes made with three accessions of the kinase insert domain mutant *InR*^353(HR)^. (E) Transheterozygotes of *InR*^E19(HR)^, *InR*^74(HR)^, and *InR*^211(HR)^. (F**)** Independent, replicate trails of transheterozygotes of *InR*^E19(HR)^, *InR*^74(HR)^, and *InR*^211(HR)^. (G**)** Transheterozygotes of *InR*^E19(HR)^, *InR*^74(HR)^, *InR*^246(HR)^ and *InR*^353(HR)^. (H**)** Independent, replicate trail of transheterozygotes of *InR*^E19(HR)^, *InR*^74(HR)^, *InR*^246(HR)^, and *InR*^353(HR)^. Life table summaries and proportional hazard statistics in Supplementary Table S4.

**Figure 7:**
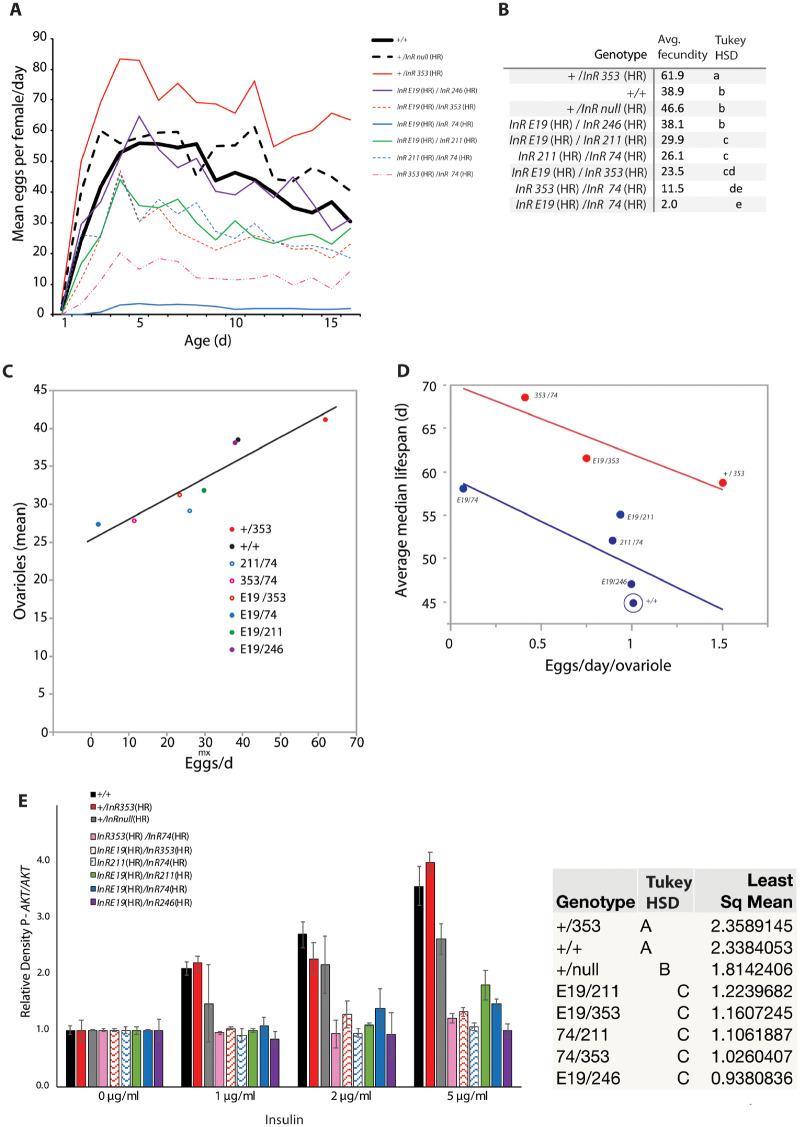
Fecundity and insulin-simulated pAkt. (A) Mean number of daily eggs laid from eclosion (age 0) through 15 days. (B) Average of daily fecundity. Genotypes not connected by same letter are significantly different, ANOVA with Tukey HSD post hoc analysis. (C) Regression of genotype mean ovariole number upon average fecundity; *R*^2^ = 0.85, *F *=* *34.1, *P *<* *0.001. (D) With *InR*^353(HR)^ as a covariate, regression of genotype lifespan (average of median lifespan is calculated among independent cohorts) and egg production per ovariole (average daily fecundity/mean ovariole number). ANCOVA: *β*_(fecundity/ovariole)_ = −9.15, *t* = −2.58, *P *=* *0.061; *β*  _(InR353)_ = 6.23, *t *=* *4.25, *P* = 0.013; *β*  _(fecundity/ovariole x InR353)_ = 1.01, *t *=* *0.28, *P *=* *0.79). (E) Quantity of phosphorylated Akt (pAKT) measured from fat body stimulated by human insulin; mean (SE) from densitometry of two independent immunoblots relative to total Akt of each sample, normalized to mean of unstimulated tissue. Two-way ANOVA; genotype, dose, and genotype-by-dose interaction, all effects *P* < 0.0001. Squares mean genotype differences not connected by same letter are significantly different (*P* < 0.05); Tukey HSD post hoc analysis.

### InR^74(HR)^: kinase domain activation loop

The activation loop (A-loop) of the *Drosophila* insulin/IGF-like receptor is homologous to that of human IR and IGF1R (Cabail *et al.* 2015). The *Drosophila* A-loop begins with a conserved DFG motif, followed by five amino acids (MTRDI), a seven amino acid sequence containing three tyrosine residues that participate in transphosphorylation (human IR: Tyr*1158*, Try*1162*, Tyr*1163*) and 13 final residues (Favelyukis *et al.* 2001). The A-loop blocks its adjacent kinase catalytic cleft in unstimulated receptors. Ligand binding induces A-loop transphosphorylation to alleviate this autoinhibition; this also provides a binding platform for substrate proteins (Hubbard *et al.* 1994; Hubbard 1997; Till *et al.* 2001). Drosophila *InR*^74(HR)^ is an Ile*1543*Phe substitution within the MTRDI motif. This change may reduce the ability for Tyr*1544* to be phosphorylated and thus diminish the domain catalytic activity or destabilize the loop binding platform (Cama *et al.* 1993; Ablooglu and Kohanski 2001; Favelyukis *et al.* 2001).

*InR* ^+(HR)^/*InR*^74(HR)^ has normal phenotypes, suggesting that the Ile*1543*Phe protomer can transactivate its complementing wild-type protomer to provide full receptor function. In contrast, only the Ile*1543*Phe protomer can be transactivated in *InR*^E19(HR)^/*InR*^74(HR)^ adults, and the lone hypomorphic function of Ile*1543*Phe increases life span. This conclusion is consistent with how *InR*^E19(HR)^/*InR*^74(HR)^ reduces insulin-stimulated kinase signaling (pAkt). To further test this explanation, we produced a double mutant where Val*810*Asp and Ile*1543*Phe were on the same protomer (*InR*^E19,74(HR)^). This allele was homozygous lethal but the genotype *InR*^+^/*InR*^E19,74(HR)^ had normal growth and lifespan. The data show that aging is slowed when receptors only activate a protomer with a Ile*1543*Phe kinase domain.

### InR^246(HR)^: kinase domain N-terminal lobe

The *InR*^246(HR)^ allele is a Val*1384*Met substitution at the invariant ATP binding loop of the N-terminal lobe (Supplementary Figure S1) (Hubbard 1997). This substitution will reduce the catalytic rate of the activated protomer (Chou *et al.* 1987; Odawara *et al.* 1989) and thus repress insulin-stimulated phosphorylation of Akt, as we observe from *InR*^E19(HR)^/*InR*^246(HR)^ fat body. The normal traits of the heterozygote *InR^+^*^(HR)^/*InR*^246(HR)^ suggests the *InR*^246(HR)^ protomer fully transactivates wild-type protomers within heterodimers. In contrast, *InR*^E19(HR)^/*InR*^246(HR)^ adults are small, as expected from their loss of kinase activity. It is unclear why this genotype does not also extend longevity although their cross-over survival pattern suggests these adults suffer high age-independent mortality that could obscure slow demographic aging.

### InR^211(HR)^: kinase domain C-terminal lobe

The Gly*1598*Arg substitution of *InR*^211(HR)^ is in the kinase domain C-terminal lobe, a site conserved in human IR (Gly*1212* Ebina) and IGF1R (Gly*1215*). These sites are three conserved residues from the substitution found in *daf-2*(e1370), the canonical IR longevity allele of *C. elegans* (Supplementary Figure S1). The homologous amino acid segment in human fibroblast growth factor receptor FGFR3 stabilizes the dimer interface during transphosphorylation (Chen *et al.* 2020). In *Drosophila* InR, the Gly*1598*Arg substitution will disrupt hydrophobic interactions among transactivating protomers. Consequently, as we observed from their limited insulin-stimulated pAkt, Gly*1598*Arg protomers will have reduced kinase catalytic activity. This molecular outcome extends lifespan, reduces growth, and limits reproduction.

### InR^353(HR)^: kinase insert domain

The *InR*^353(HR)^ allele is exceptional. The heterozygote *InR*^+(HR)^/*InR*^353(HR)^ extends lifespan yet adults have normal size and elevated fecundity. *InR*^353(HR)^ combined with *InR*^E19(HR)^ and *InR*^74(HR)^ likewise produce long-lived adults, although these are small and have reduced fecundity.

*InR* ^353(HR)^ contains the substitution Arg*1466*Cys. This arginine resides at a site found in human IRs (Arg*1092*) (Brogiolo *et al.* 2001) within in the conserved Arg-Pro-Glu sequence of the kinase insert domain (KID) (Locascio and Donoghue 2013) (Supplementary Figure S1) where Arg*1092* stabilizes kinase domain dimerization (Chen *et al.* 2020). Notably, an Arg*1092*Glu mutation in human IR produces Donohue syndrome (Takahashi *et al.* 1997). Insulin receptors with one Arg*1092*Glu substitution localize normally to cell membranes (Hall *et al.* 2020) and humans with one mutant allele appear largely normal, but individuals homozygous for this substitution have severe insulin resistance, retarded growth, and low juvenile viability (Takahashi *et al.* 1997).

*InR* ^+(HR)^/*InR^353^*^(HR)^ has wild-type insulin sensitivity, demonstrating these receptors retain kinase catalytic activity unlike other longevity assurance mutations of *InR*. How then are *InR*^+(HR)^/*InR^353^*^(HR)^ adults long-lived? The KID of *Drosophila* InR is 12 amino acids longer than the KID of human IR (Hubbard *et al.* 1994; Hanks and Hunter 1995), and contains the SH2-adaptor protein binding motif Tyr*1477*-Leu-Asn. This SH2 motif is found in mammalian IRS-2 where it binds Grb2 (Growth factor receptor-bound protein 2) (Patti *et al.* 1995) and within *Drosophila* Chico (homolog of IRS1-4) where it recruits Grb2/Drk (Grb2/Downstream-of-kinase) (Oldham *et al.* 2002). Grb2 is implicated in aging. Deletion of IRS-2 extends mouse lifespan (Taguchi *et al.* 2007; White 2014) as do null mutations of *Drosophila chico* (Clancy *et al.* 2001; Tu *et al.* 2002), and a Y*243*A substitution at the Chico SH2 site extends lifespan by repressing Grb-to-Ras signaling (Oldham *et al.* 2002; Slack *et al.* 2015).

We hypothesize the SH2 motif of the *Drosophila* InR kinase insert domain regulates lifespan through its interaction with adaptor proteins. *Drosophila* InR has been previously recognized to contain adaptor protein binding sites in the C-terminal tail and in the juxtamembrane domain (Li *et al.* 2013). Juxtamembrane domain SH2B sites recruit Lnk to modulate interactions with Chico, and mutation of *lnk* extends lifespan (Werz *et al.* 2009; Slack *et al.* 2010; Song *et al.* 2010; Almudi *et al.* 2013). We hypothesize the *Drosophila* InR kinase insert domain itself has the capacity to recruit Grb/Drk through its SH2 motif, and the Arg*1466*Cys substitution destabilizes this specific interaction to extend longevity without retarding receptor kinase activity.

### Drosophila InR domains modulate aging through distinct mechanisms

*InR^+^* ^(HR)^/*InR^353^*^(HR)^ presents a paradox: adults are highly fecund and yet long-lived. This positive association, which has been seen in other genetic manipulations of *C. elegans* and *Drosophila* (Kenyon *et al.* 1993; Gems *et al.* 1998; Jenkins *et al.* 2004), challenges a central idea of life-history theory where longevity is a trade-off with reproduction (Leroi *et al.* 2005). Our data suggest an explanation: InR regulates survival through two mechanisms (Supplementary Figure S3).

The first mechanism involves reproductive costs (Flatt 2011). Reduced germline stem cell proliferation in *Drosophila* limits the rate of egg production within ovarioles (Drummond-Barbosa 2008) and increases longevity (Flatt *et al.* 2008). Genotypes with the alleles *InR^E19^*^(HR)^, *InR^74^*^(HR)^, *InR^211^*^(HR)^ may be long-lived, in part, because their reduced egg production mitigates survival costs-of-reproduction. Genotypes with these alleles are also insulin resistant and small. We propose these growth and life history phenotypes are caused by how each amino acid substitution reduces insulin-stimulated kinase catalytic activity.

The second mechanism confers longevity assurance independent of reproductive costs. We propose the kinase insert domain recruits substrate proteins through its SH2 binding motif. The Arg*1466*Cys substitution destabilizes this interaction to induce systems that promote somatic survival while maintaining kinase catalytic activity. Thus, *InR^+^*^(HR)^/*InR*^353(HR)^ gains about 12 days of longevity. The genotypes *InR*^E19(HR)^/*InR*^353(HR)^ and *InR*^74(HR)^/*InR*^353(HR)^ also gain this reproduction-independent longevity, but now in addition to benefits conferred by reduced costs-of-reproduction.

Our explanation is consistent with how we currently understand *Drosophila chico* to regulate aging (Bohni *et al.* 1999; Clancy *et al.* 2001; Tu *et al.* 2002; Bai *et al.* 2015). As noted, Chico contains an SH2 phosphotyrosine binding site for Grb2/Drk. It also has sites to recruit p85/p60 of the PI3K complex, which regulates signaling through Akt (Oldham *et al.* 2002). Mutation of *chico* to block Grb2/Drk extends fly lifespan without reducing growth or fertility; these later traits are maintained because this mutated Chico still signals to Akt through p85/p60-PI3K (Oldham *et al.* 2002; Alic *et al.* 2014; Slack *et al.* 2015). Notably, *InR*^353(HR)^ extends fly lifespan without reducing growth or fertility and maintains its ability for insulin to stimulate Akt phosphorylation. *Chico* mutated at the p85/p60 sites, on the other hand, extends life span but represses growth and reproduction. This suite of traits is seen in genotypes with the alleles *InR^E19^*^(HR)^, *InR^74^*^(HR)^, and *InR^211^*^(HR)^, which also do not phosphorylate Akt in response to insulin.

The *Drosophila* IR appears to regulate aging through distinct systems modulated through structure-defined domains. This view reflects an emerging concept whereby protein tyrosine receptors control varied phenotypes through the action of specific protein interactions regulated by structural domains (Oldham and Hafen 2003; Liu *et al.* 2012; Li *et al.* 2013; Zinkle and Mohammadi 2018). Future biochemical and genetic studies will test our mechanistic hypotheses.
